# Gut microbial community plasticity as a climate shield mediating sea cucumber resilience to ocean acidification and warming

**DOI:** 10.1093/ismeco/ycaf188

**Published:** 2025-10-23

**Authors:** Encui Shan, Zhenglin Yu, Xiao Cong, Chaowei Hou, Xueying Guo, Lei Pang, Jianmin Zhao, Qing Wang, Xiutang Yuan

**Affiliations:** Research and Development Center for Efficient Utilization of Coastal Bioresources, Yantai Institute of Coastal Zone Research, Chinese Academy of Sciences, Yantai 264003, Shandong Province, P. R. China; Laboratory for Marine Biology and Biotechnology, Qingdao Marine Science and Technology Center, Qingdao 266000, Shandong Province, P. R. China; University of Chinese Academy of Sciences, Beijing 100049, P.R. China; Research and Development Center for Efficient Utilization of Coastal Bioresources, Yantai Institute of Coastal Zone Research, Chinese Academy of Sciences, Yantai 264003, Shandong Province, P. R. China; Muping Coastal Environment Research Station, Yantai Institute of Coastal Zone Research, Chinese Academy of Sciences, Yantai 264003, Shandong Province, P. R. China; Research and Development Center for Efficient Utilization of Coastal Bioresources, Yantai Institute of Coastal Zone Research, Chinese Academy of Sciences, Yantai 264003, Shandong Province, P. R. China; Muping Coastal Environment Research Station, Yantai Institute of Coastal Zone Research, Chinese Academy of Sciences, Yantai 264003, Shandong Province, P. R. China; Research and Development Center for Efficient Utilization of Coastal Bioresources, Yantai Institute of Coastal Zone Research, Chinese Academy of Sciences, Yantai 264003, Shandong Province, P. R. China; Laboratory for Marine Biology and Biotechnology, Qingdao Marine Science and Technology Center, Qingdao 266000, Shandong Province, P. R. China; University of Chinese Academy of Sciences, Beijing 100049, P.R. China; Muping Coastal Environment Research Station, Yantai Institute of Coastal Zone Research, Chinese Academy of Sciences, Yantai 264003, Shandong Province, P. R. China; Research and Development Center for Efficient Utilization of Coastal Bioresources, Yantai Institute of Coastal Zone Research, Chinese Academy of Sciences, Yantai 264003, Shandong Province, P. R. China; Laboratory for Marine Biology and Biotechnology, Qingdao Marine Science and Technology Center, Qingdao 266000, Shandong Province, P. R. China; University of Chinese Academy of Sciences, Beijing 100049, P.R. China; Research and Development Center for Efficient Utilization of Coastal Bioresources, Yantai Institute of Coastal Zone Research, Chinese Academy of Sciences, Yantai 264003, Shandong Province, P. R. China; Laboratory for Marine Biology and Biotechnology, Qingdao Marine Science and Technology Center, Qingdao 266000, Shandong Province, P. R. China; University of Chinese Academy of Sciences, Beijing 100049, P.R. China; Research and Development Center for Efficient Utilization of Coastal Bioresources, Yantai Institute of Coastal Zone Research, Chinese Academy of Sciences, Yantai 264003, Shandong Province, P. R. China; University of Chinese Academy of Sciences, Beijing 100049, P.R. China; Muping Coastal Environment Research Station, Yantai Institute of Coastal Zone Research, Chinese Academy of Sciences, Yantai 264003, Shandong Province, P. R. China; Research and Development Center for Efficient Utilization of Coastal Bioresources, Yantai Institute of Coastal Zone Research, Chinese Academy of Sciences, Yantai 264003, Shandong Province, P. R. China; University of Chinese Academy of Sciences, Beijing 100049, P.R. China; Muping Coastal Environment Research Station, Yantai Institute of Coastal Zone Research, Chinese Academy of Sciences, Yantai 264003, Shandong Province, P. R. China; Research and Development Center for Efficient Utilization of Coastal Bioresources, Yantai Institute of Coastal Zone Research, Chinese Academy of Sciences, Yantai 264003, Shandong Province, P. R. China; Muping Coastal Environment Research Station, Yantai Institute of Coastal Zone Research, Chinese Academy of Sciences, Yantai 264003, Shandong Province, P. R. China

**Keywords:** climate change, *Apostichopus japonicus*, gut microbiome, metabolomic profiling, metabolic plasticity

## Abstract

Ocean acidification (OA) and ocean warming (OW) pose escalating threats to marine ecosystems, particularly to benthic organisms, such as sea cucumbers, that play pivotal roles in nutrient cycling and sediment health. Existing research mainly addresses sea cucumbers’ physiological responses, overlooking gut microbial communities and metabolites in their stress adaptation. Herein, a mesocosm was constructed and analyzed by using integrated gut microbiome and metabolomics approaches to investigate the responses of sea cucumbers *Apostichopus japonicus* to OA and OW. Results revealed that microbial community plasticity underpins holobiont adaptation, with warming restructuring gut microbiota toward thermotolerant taxa, whereas acidification enriches alkalinity-modulating Rhodobacteraceae and *Halioglobus* sp. Metabolomic profiling identified 43 amino acid derivatives with significantly increased concentrations in OA and OW groups, including upregulated N-methyl-aspartic acid and γ-glutamyl peptides that stabilize macromolecules and enhance redox homeostasis. Conversely, antioxidative metabolites (e.g., ergothioneine, L-homocystine) are suppressed, reflecting trade-offs between energy allocation and stress protection. In OW group, the antioxidant synthesis pathway is shifted to energy metabolism related to heat tolerance, whereas in OA group, energy is preferentially used for alkalinity regulation pathways rather than oxidative stress defense. Changes in microbial community structure mechanistically explain the trends in metabolite concentrations, as the proliferation of *Vibrio* spp. in the OW group drives lysine catabolism, leading to a significant increase in L-saccharopine levels. Bacteroidetes reduction in the OA group correlates with L-homocystine downregulation, suggesting that pH-driven microbial interactions are disrupted. These findings demonstrate gut microbiota reshape community structure and metabolism to mitigate synergistic climate stress, emphasizing microbiome-mediated resilience in marine ecosystems amid global climate change.

## Graphical Abstract

**Graphical Abstract:**
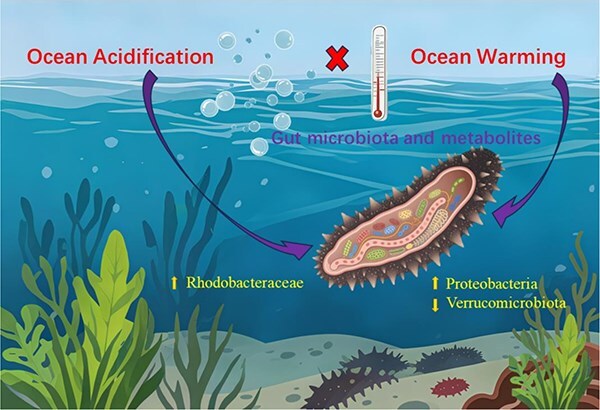

## Introduction

The associations between eukaryotes and microorganisms are ubiquitous, and host-associated microbial communities play a pivotal role in host development, health, immunity, and environmental interactions [1–3]. The tight relationships between hosts and associated microbial have even inspired the hologenome evolution theory, which posits that hosts and their associated microbiota should be regarded as a single unit of selection (termed a “holobiont”), emphasizing that microbial symbionts as key contributors to host fitness [4]. In recent years, studies exploring the taxonomic and functional diversity of host-associated microbial communities have gained increasing attention, particularly in the context of marine invertebrates [1, 5, 6]. The tissues of marine invertebrates typically harbor species-rich microbial communities, among which the gut microbiome stands out as a primary site of host–microbe interaction, playing a key role in mediating metabolic regulation, nutrient absorption, and immune defense [7–9]. Thus, the stability of this host–microbe interaction has become increasingly vulnerable to the rapid oceanic changes driven by human activities.

The oceans have absorbed ~25% of anthropogenic atmospheric CO₂ emissions, resulting in a progressive increase in CO₂ dissolution and a concomitant decrease in seawater pH [10–12]. Meanwhile, sustained anthropogenic CO₂ emissions also contribute to global warming, which elevates seawater temperatures. These two outcomes of anthropogenic activity have precipitated two major oceanic phenomena, namely, ocean acidification (OA) and ocean warming (OW), which pose substantial threats to marine ecosystems [11, 13–15]. Notably, OA and/or OW not only affect marine invertebrate’s physiological status but also induce changes in their associated gut microbial communities. Studies have shown that rapid environmental changes over short timescales can disrupt gut microbiota [16]. For example, OA can reduce the relative abundance of probiotic bacteria in the gut of *Crassostrea gigas*, which facilitates the proliferation of pathogenic bacteria and increases oysters’ susceptibility to pathogens [17]. OW exerts a more pronounced impact on the gut microbiota, such as the reduction in the abundance of Actinobacteria in sea urchins exposed to high temperatures, which may impair their ability to resist pathogenic infections [18]. High water temperatures can disrupt the microbial community of *Seriola dumerili*, wherein increased abundances of *Psychrobacter*, *Chryseomicrobium*, and *Enterovibrio* serve as biomarkers of ecological dysbiosis [19]. Such temperature-induced microbial imbalances may further lead to the proliferation of pathogenic bacteria, such as *Vibrio* spp*.*, consequently elevating disease susceptibility in hosts [17, 20, 21].

Studies have shown that the effects of OA and OW on highly calcifying taxa have been extensively investigated (e.g. corals and bivalves) [15, 22–25]. While research on benthic invertebrates with low calcification capacities has advanced—with studies documenting their physiological and ecological responses to climate stressors—these studies remain unevenly distributed across taxonomic groups and research foci [26–29]. The attention to the mechanistic roles of gut microbiota in mediating host acclimation to the stress of OA and/or OW remains limited. Even for taxa where OA/OW-related gut microbiota research has emerged, investigations often lack exploration of mechanisms or metabolic analyses—yet metabolic analysis serves as the core bridge connecting gut microbial community structure to host physiological functions and environmental adaptation [30, 31]. This limitation leaves critical gaps in our understanding of how gut microbiota, together with their metabolites, support host adaptation to OA and OW stress.

In this study, we selected the sea cucumber (*A. japonicus*)—a typical low-calcifying benthic invertebrate—as the research subject, with a focus on characterizing the variation patterns of its gut microbiota and metabolites under OA and/or OW. As a key benthic functional group in coastal areas, sea cucumbers can promote organic matter decomposition and nutrient recycling through bioturbation, thereby maintaining the health and productivity of benthic ecosystems [32, 33]. Nowadays, research on the effects of OA and OW of sea cucumbers has primarily focused on physiological responses. Studies have demonstrated that low pH exposure reduces energy intake, leading to decreased somatic growth allocation, whereas elevated temperatures alter protein dynamics in respiratory trees [34–37]. However, these organism-level findings remain disconnected from potential microbe-mediated mechanisms, leaving a fundamental gap in our understanding of how host-associated microbiota contribute to resilience in changing ocean conditions. Crucially, the role of the gut microbiome in facilitating environmental adaptation, particularly through metabolic compensation or stress response modulation, remains virtually unexplored despite the growing recognition of host–microbiome interactions in other marine ectotherms. Herein, we address this knowledge gap by integrating microbial community structure with metabolic function, elucidating how microbes enhance host stress resistance by modulating metabolite changes. This research aims to (i) analyze the structural and functional changes in the gut microbiome under OA and/or OW comprehensively and (ii) assess the potential ecological role of temperate sea cucumbers in future climate change scenarios. How the gut microbiota of sea cucumbers supports host adaptation through community restructuring and metabolic adjustments under multiple stressors remains a critical scientific question. Addressing this gap will not only help uncover the survival strategies of sea cucumbers under environmental stress but also shed light on microbiome-mediated mechanisms that support sediment health, carbon turnover, and benthic ecosystem resilience. These insights may inform ecosystem-based management and marine conservation strategies under future climate change scenarios.

## Materials and methods

### Sediment collection, mesocosm construction, and animal preacclimation

A box corer (25 cm × 25 cm) was employed to collect intact sediment cores from the nearshore area of Yantai City, China (Fig. S1.A), in May 2024. Surface sediments (0–7 cm depth) were subsampled by using an acrylic sheet and transferred undisturbed into custom-made tanks (78 L, 65 cm × 40 cm × 30 cm). Each tank contained 75 L filtered seawater and was equipped with a submersible pump (Sunsun, JP-064, 800 L/h), with a continuous circulation cycle (24 h/day) to ensure water change. Each tank contained three random sediment cores, with excess sediment collected as a food source for experimental animals during acclimatization to minimize variability. Sediments were transported to the laboratory within 2 h and covered with shading nets to mitigate temperature fluctuations [38].

In the laboratory, the sediment cores were gradually infused with local seawater (13.6°C) to prevent resuspension then aerated in a mesocosm. A submersible pump (Sunsun, JP-064, 800 L/h) was installed to maintain water circulation and prevent sediment anoxia [39]. The system was acclimated at ambient temperature for 26 days to re-establish internal geochemical gradients within the sediments [40], with all cores maintained under consistent conditions.

Experimental *A. japonicus* individuals were obtained from a local farm and acclimatized in the laboratory for two weeks. During this period, they were fed with a mixture of dried sediment and sargassum powder (3:1 ratio) once daily. Twenty-four individuals (39.8 ± 3.3 g) exhibiting good morphology, complete body condition, and normal feeding behavior were selected for the experiment. Prior to the study, the animals were allowed to empty their guts, and excreta were promptly removed to prevent reingestion. For the experiment, the 24 selected individuals were allocated to replicate tanks, with 2 individuals per tank to simulate their natural density in the wild [33]. Rocks with attached *Ulva lactuca* leaves were collected, washed with filtered seawater to remove epiphytic organisms, and placed in each mesocosm to simulate the natural habitat of *A. japonicus*. Throughout the experimental period, no additional food was supplied to the mesocosms, and the animals were exclusively fed on natural sediments and algal tissue (Fig. S1.B).

### Experimental design and sample collection

The experimental design was based on the scenario of the IPCC Sixth Assessment Report (2021) SSP5–8.5, which projects that under high greenhouse gas emissions, ocean *p*CO₂ will rise to 1000–1135 ppm by the end of the 21st century, corresponding to a decline in ocean pH to ~7.77 (IPCC, 2021). This scenario was used to simulate future OA conditions in our experiment. CO_2_ (99.99%) was introduced into the mesocosms to reduce the water pH gradually by 0.05 units per day over one week until it stabilized at 7.77 (mean ± SD = 7.77 ± 0.02). The low-pH environment was maintained with an online digital pH sensor (Y532-A) and acidification monitoring system. The system operated via a closed-loop feedback mechanism, where target pH was pre-input, and the pH sensor continuously measured seawater pH in each mesocosm at every 2 seconds intervals, transmitting real-time data to the system. If the measured pH exceeded the target value, the system automatically increased CO₂ bubbling flow rate to lower pH, and the reverse occurred accordingly. This setup ensured pH stability within ±0.02 of the target value throughout the experiment. In contrast, the control (Con) and OW (warming only) groups were held at ambient seawater pH, with pH also monitored daily using the same sensor to confirm no unintended fluctuations. Water temperature was regulated by employing a heating rod (Jiabao, 50 W), with the temperature of the experimental group following natural nearshore variations, ensuring a constant 2°C difference between warming and no-warming groups. The study comprised four experimental groups, namely, the Control (Con), OW, OA, and OA + Warming (OAW), each with three replicates (*n* = 3). The formal experiment lasted 60 days, a duration that is sufficient to observe detectable changes in microbial communities [39].

Water samples were drawn from each mesocosm every week by using a sterile syringe to assess changes in the mesocosm carbonate system. Dissolved inorganic carbon (DIC) was measured in filtered samples (0.45 μm membrane) transferred to 40 mL borosilicate glass bottles (bubble-free). A total of 50 μl of saturated HgCl_2_ (0.02%–0.05% by volume) was added to the water samples immediately after collection to inhibit microbial activity, and samples were stored in the dark at 4°C until analysis [41]. DIC concentrations were measured using a DIC analyzer (AS-C3, Apollo SciTech Inc.) calibrated with standard seawater (Dikson Laboratory, Batch 216, 2029.63 ± 0.28 μmol/kg). To calculate additional carbonate system parameters—including total alkalinity (TAlk), partial pressure of carbon dioxide (*p*CO₂), aragonite saturation state (Ωarag), and DIC components (HCO₃^−^, CO₃^2−^, and free CO₂)—the CO₂SYS.XLS program (version 24) was employed [41], with input variables consisting of the measured DIC, pH, and temperature values for each experimental group. This program derives carbonate system parameters from two known variables (here, DIC and pH) under specified temperature and pressure conditions by solving the chemical equilibrium equations governing the seawater carbonate system using the selected equilibrium constants [42].

### Deoxyribonucleic acid extraction and 16S ribosomal ribonucleic acid sequencing

After the 60-day incubation period, sea cucumbers from each group were surface-disinfected with 70% ethanol. The intestinal tract was aseptically extracted, and only the contents of the mid-hindgut were collected for subsequent analysis. This selection was based on prior studies on *A. japonicus* demonstrating that the mid-hindgut is the primary site of stable host–microbe interactions and functional metabolite accumulation [43, 44]. Then the mid-hindgut contents immediately frozen in liquid nitrogen, and stored at −80°C. Microbial deoxyribonucleic acid (DNA) was extracted from the intestinal samples by using HiPure Fecal DNA Kit (Magen, Guangzhou, China) in accordance with the manufacturer’s instructions. DNA integrity was verified through 1% agarose gel electrophoresis. The V3–V4 hypervariable region of the bacterial 16S ribosomal ribonucleic acid (rRNA) gene was amplified by using primers 341F (CCTACGGGNGGCWGCAG) and 806R (GGACTACHVGGGTATCTAAT) [45, 46]. Polymerase chain reaction (PCR) was performed with a 25 μl mixture containing 15 μl of Phusion High-Fidelity PCR Master Mix, 0.2 μm primers, and 10 ng of the DNA template. Thermal cycling conditions included an initial denaturation cycle at 98°C for 1 min, followed by 30 cycles of denaturation at 98°C (10 s), annealing at 50°C (30 s), and extension at 72°C (30 s), with a final extension at 72°C for 5 min. PCR products were purified by employing magnetic beads, and equimolar pooling was performed on the basis of concentration measurements. Target bands were recovered, and libraries were constructed. Library quality was assessed through Qubit quantification and quantitative PCR amplification. Only libraries meeting quality control criteria were selected for sequencing. After obtaining raw sequencing data, rarefaction was performed to control for differences in sequencing depth among groups, and the rarefaction curves have been attached in Fig. S2 for reference. Raw data have been deposited to National Center for Biotechnology Information (NCBI) under the BioProject number PRJNA1270743.

### Microbiome data processing

After barcode and primer sequences were removed, reads from each sample were merged by using FLASH (version 1.2.11; http://ccb.jhu.edu/software/FLASH/) [47] to generate raw tags. Reverse primer sequences were then matched and trimmed by using Cutadapt (version 3.3) to eliminate potential interference in downstream analyses. High-quality clean tags were obtained by filtering the merged raw tags with fastp (version 0.23.1) [48]. Chimeric sequences were identified and removed by aligning the resulting tags against reference databases (Silva database, https://www.arb-silva.de/ for 16S/18S; Unite database, https://unite.ut.ee/ for ITS), yielding effective tags [49]. The effective tags were denoised by using the DADA2 module in QIIME2 to generate amplicon sequence variants (ASVs) and feature tables [50]. The taxonomic classification of ASVs was performed by using QIIME2 with the Silva 138.1 database, and a phylogenetic tree was constructed on the basis of ASV sequences. The relative abundance distribution of the top 10 species at the phylum and family levels was visualized by using histograms. Species diversity indices (Chao1, Pielou_e, Shannon, Simpson) were calculated by using QIIME2, then Tukey’s test was used to analyze whether there were significant differences in species diversity among groups. Nonmetric multidimensional scaling (NMDS) analysis was conducted in R with the ade4 and ggplot2 packages. Community structure differentiation was assessed through ANOSIM statistical analysis, and biomarkers across treatment groups were identified by using LEfSe. To assess the similarity of gut microbial communities among different groups, weighted UniFrac distance matrices were calculated using QIIME2 based on the ASV table. UPGMA (Unweighted Pair-Group Method with Arithmetic Means) clustering analysis was then performed on these distance matrices using the PGMA.tre function in R software. Potential functional profiles were predicted and analyzed using Tax4Fun (V0.3.1) in R based on the 16S Silva database.

### Metabolite extraction and LC–MS

A mixture of 136 amino acid standards (Sigma-Aldrich) was prepared with a concentration of 5 mg/mL to generate a linear calibration stock solution supplemented with an isotope internal standard (IS) solution (Standaro, Shanghai) at a defined concentration. The IS solution was thoroughly mixed. A 20 μl aliquot of intestinal contents was combined with 80 μl of precipitant (acetonitrile:methanol = 1:1) containing the mixed IS, vortexed, and sonicated for 10 min. The mixture was incubated at −20°C for 60 mins and centrifuged at 12000 rpms (4°C) for 15 min. The supernatant was collected for LC–MS. Amino acids were quantified by using an ultrahigh-performance liquid chromatography–tandem mass spectrometry system (ExionLC™AD UHPLC-QTRAP 6500+, AB SCIEX Corp., Boston, MA, the USA) (Novogene, China). Separation was achieved on an ACQUITY UPLC BEH amide column (2.1 × 100 mm, 1.7 μm) with a mobile phase consisting of 5 mM ammonium acetate containing 0.1% formic acid (solvent A) and acetonitrile with 0.1% formic acid (solvent B) delivered at 0.30 mL/min. The gradient program was as follows: 90% B (0 min), 90%–85% B (2.0 min), 85%–75% B (3.5 min), 75%–70% B (7.0 min), 70%–45% B (10 min), 45%–90% B (11.1 min), and 90% B (13 min). The mass spectrometer was operated in positive ion mode with an electrospray ionization source under the following parameters: ion source temperature of 550°C, ion spray voltage of 5500 V, curtain gas of 35 psi, nebulizer gas of 50 psi, and auxiliary gas of 60 psi. Multiple reaction monitoring was employed for data acquisition. Method validation included linearity verification by using a concentration series of standard solutions; the determination of the limit of quantification via the signal-to-noise ratio method (10:1); and the evaluation of precision (intraday and interday RSD ≤ 15%, *n* = 3), accuracy (target analyte recovery 85%–115%), and stability (RSD of target analytes ≤15% over 24 h).

### Metabolomics data processing

Targeted amino acid metabolomics analysis was conducted to quantify and compare the concentrations of amino acid metabolites across experimental conditions with the aim of elucidating the potential effects of environmental changes on nitrogen metabolism by gut microbiota. Differential metabolites were screened on the basis of the following criteria: (i) *P* < .05, as determined by one-way ANOVA, indicating significant concentration differences between the Con and treatment groups; (ii) fold change (FC) > 1.2 or < 0.8, reflecting substantial concentration variations between groups; and (iii) variable importance in projection score > 1 in the orthogonal partial least squares-discriminant analysis (OPLS-DA) model, highlighting metabolites with significant contributions to group discrimination [51]. If no differential metabolites were identified on the basis of the above criteria, then the analysis focused solely on the functional roles of the metabolites themselves. Multivariate statistical analyses, including principal component analysis (PCA) and OPLS-DA, were performed by using MetaboAnalyst 5.0 to identify metabolic patterns and discriminate between treatment groups. Univariate statistical analyses, such as Student’s t-test or one-way ANOVA (*P* < .05), were applied to assess significant differences in amino acid concentrations among groups.

## Results

### Parameters of the mesocosm carbonate system

**Figure 1:**
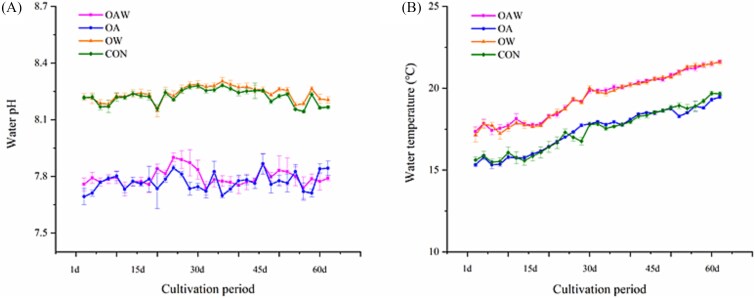
The dynamic variations in seawater pH (A) and temperature (B) across different groups during the cultivation period.

The seawater temperature and CO_2_ concentration have been successfully maintained within the set parameters. Table 1 shows that the pH of the ambient CO_2_ group (Con and OW) remained between 8.23 and 8.24, whereas that of the elevated CO_2_ treatment groups (OA and OAW) remained stable at approximately 7.77 (Fig. 1A). The ambient temperature groups (Con and OA) presented a mean temperature of approximately 17.4°C and temperature range of 15.65°C–19.49°C, whereas the elevated temperature groups (OW and OAW) exhibited a mean temperature of 19.4°C and temperature range of 17.50°C–21.50°C. Consequently, a consistent temperature difference of approximately 2°C was maintained between the ambient and elevated temperature groups throughout the entire experimental period (Fig. 1B). Additional hydrological and carbonate parameters in the experiment are presented in Table 1.

**Table 1:** Hydrographic and carbonate parameters of experimental groups during the cultivation period.

Treatment	CON	OW	OA	OAW
Measured pH_NBS_	8.23 ± 0.03	8.24 ± 0.04	7.77 ± 0.04	7.79 ± 0.04
Temp (°C)	17.43 ± 1.32	19.45 ± 1.43	17.42 ± 1.28	19.48 ± 1.39
Salinity	32.37 ± 0.56	32.90 ± 0.66	32.34 ± 0.46	32.79 ± 0.51
DIC (μmol/kg)	2551 ± 54	2604 ± 84.9	2779 ± 106	2817 ± 110
Calculated TAlk (μmol/kg)	2841 ± 61	2933 ± 103	2848 ± 102	2911 ± 110
*p*CO_2_ (μatm)	430.37 ± 35.20	430.04 ± 39.52	1441.03 ± 168.77	1379.24 ± 156.22
Ω_Ca_	5.47 ± 0.46	6.18 ± 0.70	2.15 ± 0.22	2.52 ± 0.29
Ω_Ar_	3.51 ± 0.30	3.99 ± 0.47	1.38 ± 0.14	1.63 ± 0.19

Note: All data are presented as mean ± SD (*n* = 3).

### Gut microbiome composition and functional characterization in *Apostichopus japonicus*

**Figure 2:**
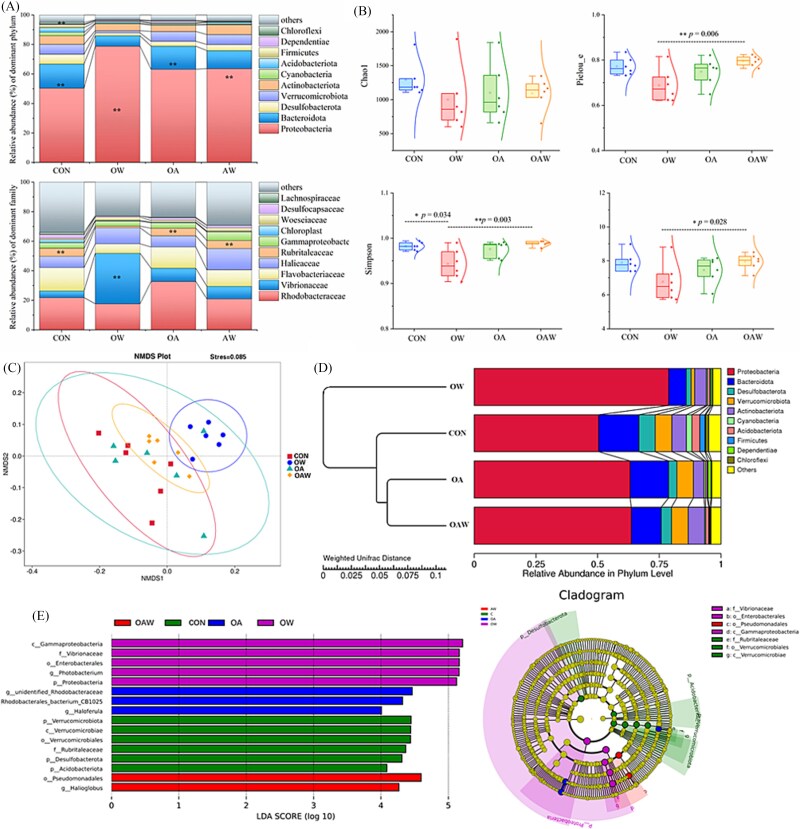
Microbial community compositions and diversities in the gut of *A. japonicus*. (A) Relative abundance of the top 10 species at the phylum and family levels in gut microbiota. (B) Diversity of gut microbiota in different treatment groups. (C) NMDS analysis was used to distinguish differences within and between community groups. (D) UPGMA clustering tree based on weighted UniFrac distances. (E) LEfSe identified the biomarkers with statistically significant differences between groups (left). Distinct colors in the cladogram represent microbial taxa that play important roles in their respective groups (right).

The structure and potential functional profiles of the gut microbiome of *A. japonicus* were analyzed under the effects of OA or/and OW. Dominant microbial taxa at the phylum and family levels were visualized in Fig. 2. At the phylum level, OW treatment significantly increased the relative abundance of Proteobacteria (F _[3, 20]_ = 8.414, *P* < .001), but significantly reduced that of Verrucomicrobiota (F_(3,20)_ = 7.738, *P* = .01). Chloroflexi in all treatment groups significantly reduced relative to that in the Con group (Fig. 2A, *P* < .05, Table S1). At the family level, OW treatment significantly increased the relative abundance of Vibrionaceae (F _[3, 20]_ = 14.558, *P* < .001), but significantly reduced that of Rubritaleaceae (Fig. 2A, F _[3, 20]_ = 7.239, *P* = .01, Table S2). Gut microbial α diversity indices, such as the Pielou, Shannon, and Simpson indices, were significantly lower under the OW treatment compared with other treatments (Fig. 2B, *P* < .05). NMDS analysis and PERMANOVA showed that in terms of microbial community distribution, OW treatment separated from other treatments (Fig. 2C and Table S3, *P* < .05). UPGMA clustering based on weighted UniFrac distances revealed distinct clustering patterns among treatment groups (Fig. 2D). Specifically, samples from the same treatment tended to cluster together, indicating consistent community structures within groups. Notably, the OW group with the largest average weighted UniFrac distance from all other groups (Fig. 2D).

LEfSe analysis was performed to identify statistically significant biomarkers among groups. It revealed that distinct microbial taxa were associated with each experimental condition. In the OW group, Gammaproteobacteria, Vibrionaceae, Enterobacteriales, and *Photobacterium* were identified as significant biomarkers. The OA group was characterized by Rhodobacterales, including Rhodobacteraceae and *Rhodobacter*, as well as *Haloferula* from Actinobacteria. The Con group exhibited biomarkers, including Verrucomicrobiae, Rubritaleaceae, Desulfobacterota, and Acidobacteriota. The OAW group was associated with Pseudomonadales and *Halioglobus* as key biomarkers (Fig. 2E).

**Figure 3:**
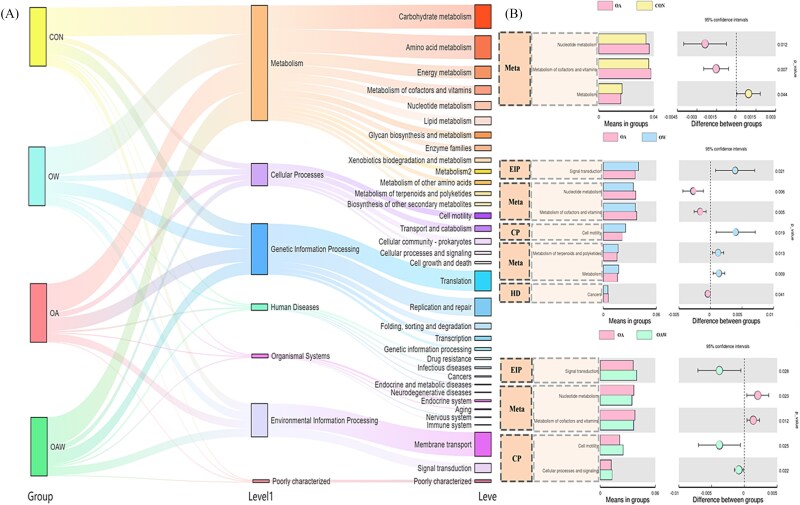
Functional annotation of gut microbiota of *A. japonicus*. (A) Sankey diagram of predicted functional type assignments for *A. japonicus* gut microbiota. (B) Comparative analysis of functional type differences versus the con group. The abbreviated represent: Meta (metabolism), EIP (environmental information processing), CP (cellular processes), and HD (human diseases).

The functional profiles of gut microbial community across four groups were visualized via the Sankey diagram, revealing distinct distributions in hierarchical functional levels (Fig. 3A). At the KEGG Level 1 functional hierarchy, metabolic pathways dominated across all treatment groups, primarily comprising carbohydrate metabolism, amino acid metabolism, and energy metabolism, etc. Differential analysis of enriched community functions revealed that the OA group exhibited statistically significant functional divergence compared to the other three groups (Fig. 3B, *P* < .05).

### Association analysis of gut–sediment microbiota

**Figure 4:**
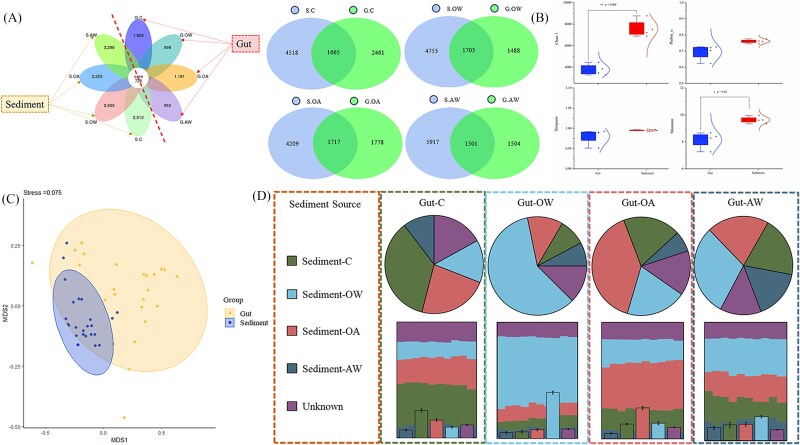
Combined analysis of sediment and *A. japonicas* gut microbiota. (A) Unique and shared characteristic sequences of sediment and gut microbiota. (B) Comparison of α diversity between sediment and gut microbiota communities. (C) Heatmap of the β diversity index matrix based on unweighted UniFrac distances. (D) NMDS analysis of sediment and gut microbiota distribution. (E) Bayesian model–based traceability analysis of gut microbiota.

Given the critical role of sediment as a reservoir of microbial diversity and a potential source of the gut microbiota of *A. japonicus*, sequencing and analyzing sediment samples is essential to elucidate microbial exchange between the environment and host. The number of shared/unique characteristic sequences in the sediment samples was significantly higher than that in the gut of *A. japonicas* (Fig. 4A). Moreover, the Chao1 and Shannon indices of microbial communities in sediments were significantly higher than those in gut microbial communities (*P* < .05, Fig. 4B, Table S4). NMDS analysis was conducted on sediment and gut microbiota communities, and the results showed that the distribution of gut microbiota was similar to that of sediment communities, suggesting that the microbial communities in the gut are primarily derived from sediment (Fig. 4C). Furthermore, the findings of the Bayesian model–based source analysis of gut microbiota aligned with the predicted results, demonstrating that gut microbiota predominantly originates from sediments (Fig. 4D).

### Differential analysis of amino acid metabolites in gut microbiota

**Figure 5:**
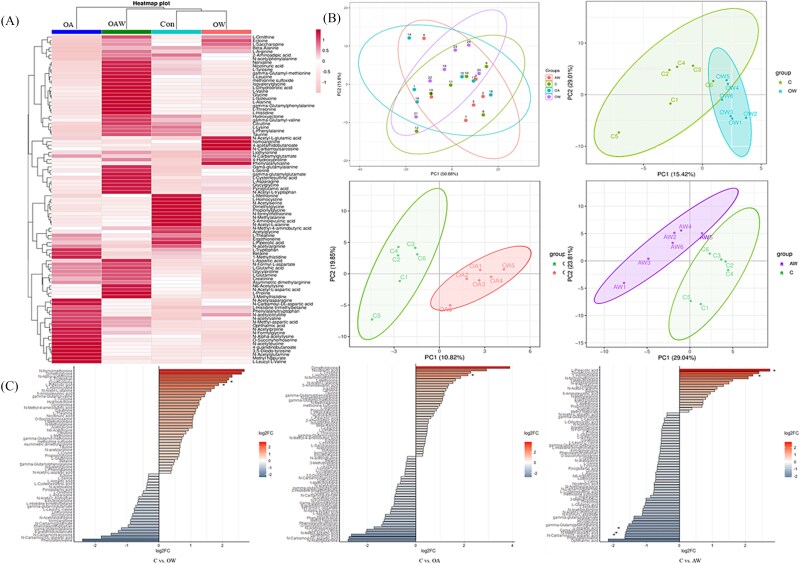
Enrichment and differential analysis of gut microbial metabolites. (A) Cluster heatmap of all amino acid metabolites in different groups. (B) PCA between treatment groups and PLS-DA between the con group and other groups. (C) Screening of differential amino acid metabolites. The right and left part indicate the upregulation or downregulation of metabolite content, respectively, and * represents metabolites with significant differences selected.

Amino acid metabolism plays a pivotal role in the physiological functions of microorganisms and hosts, acting as a key mediator in microbial–host interactions. We investigated the amino acid metabolites derived from gut microbiota, identifying 91 distinct metabolites (Table S5). These metabolites were systematically classified into the following categories on the basis of their chemical properties, metabolic pathways, or biological functions: essential amino acids and their derivatives (e.g. L-alanine, L-valine, L-leucine, and L-proline); nonproteinogenic amino acids with important metabolic functions (e.g. β-alanine, γ-aminobutyric acid, and L-ornithine); intermediates in amino acid biosynthesis or catabolism (e.g. pyroglutamic acid, 2-aminoadipic acid, and 4-acetamidobutanoate); acetylated or methylated amino acid derivatives (e.g. *N*-acetyl-L-alanine and *N*-acetyl-L-glutamic acid); dipeptides and oligopeptides (e.g. glycylglycine and L-leucyl-L-valine); amino acid–related small molecules involved in energy metabolism or cellular signaling (e.g. betaine, creatinine, and taurine); terminal products or excretory metabolites of amino acid metabolism (e.g. creatinine, uric acid, and L-dihydroorotic acid); and specialized amino acid metabolites with unique structural or functional properties (e.g. 3,5-diiodo-tyrosine, liothyronine, and L-theanine). The clustering heatmap revealed differences in amino acid metabolite content and enrichment among groups (Fig. 5A). For example, the levels of ergothioneine, L-homocystine, L-pipecolic acid, and L-methionine in the Con group were significantly elevated compared with those in the other three groups. In the OW group, only L-saccharopine showed a high concentration. In the OA group, the concentrations of L-aspartic acid, L-leucyl-L-valine, *O*-succinyl homoserine, and *N*-methyl-aspartic acid surpassed those in the other groups. The OAW group displayed a great variety of metabolites with high levels. These metabolites primarily included L-glutamic acid, nicotinuric acid, methionine sulfoxide, L-tyrosine, and L-glutamine. PCA and PLS-DA were performed to characterize intergroup differences. PCA revealed no clear separation between groups, whereas PLS-DA identified metabolites that differed between the treatment and Con groups, further confirming that acidification and warming stressors substantially influenced metabolic profiles (Fig. 5B). Deviation bar plots were generated on the basis of the metabolites showing significant differences between the Con and treatment groups (Fig. 5C and Table S6). Compared with the Con group, the OW treatment group exhibited a marked reduction in the levels of L-pipecolic acid and ergothioneine, whereas the OA group showed a marked decrease in L-homocystine. Similarly, the OAW group displayed notably lower levels of L-pipecolic acid and L-homocystine but a pronounced increase in L-aspartic acid, *N*-carbamoyl-DL-aspartic acid, and *N*-methyl-aspartic acid (Fig. 5C).

## Discussion

### Microbial community responses to environmental stressors

The gut microbiome, which acts as a virtual endocrine organ, plays a pivotal role in mediating host–environment interactions through nutrient metabolism, immune regulation, and microbial community dynamics [52]. Our findings reveal that that OW induces distinct shifts in the gut microbiota structure of *A. japonicus*, with notable increases in the relative abundance of Proteobacteria and concurrent declines in Verrucomicrobiota. A key component of this shift is the enhanced proliferation of Vibrionaceae under elevated temperatures—a pattern consistent with broader ecological observations that warming favors the expansion of opportunistic microbial taxa, including potential pathogens [53, 54]. Such proliferation may compromise host fitness under chronic heat stress. While the direct implications of this proliferation for *A. japonicus* health require further investigation, the accompanying reduction in microbial α diversity and clear separation of the OW microbial community in ordination analyses suggest that thermal stress disrupts gut microbiome stability. Such instability may compromise the microbiome’s ability to provide consistent functional support (e.g. nutrient degradation, immune modulation) to the host, thereby weakening the holobiont’s capacity to buffer environmental fluctuations.

OA also drives substantial restructuring of the gut microbiome, which was characterized by the replacement of 38% of the core microbial taxa with Rhodobacteraceae. Rhodobacteraceae are well-documented for their role in dimethylsulfoniopropionate (DMSP) metabolism—a function that generates climate-active gasses and supports carbon cycling in coastal ecosystems [55, 56]. The enrichment of a taxon with a functionally critical role in carbon cycling provides indirect evidence that, by maintaining this key metabolic function under acidification, the gut microbiome may help offset OA-induced disruptions to the host’s nutrient acquisition or sediment-processing roles. The synergistic effects of OAW further amplify these microbiome shifts, leading to the emergence of unique microbial biomarkers not observed under single stressors—such as *Halioglobus*, a genus within the *Rhodobacteraceae* family. This finding—coupled with prior studies documenting *Halioglobus*’ capacity to tolerate fluctuating pH and temperature, and to modulate alkalinity in coastal sediment environments—suggests a potential trajectory of microbial community restructuring toward taxa with documented capacities to cope with multistressor conditions, albeit with the caveat that this may occur at the cost of reduced functional redundancy [57].

Beyond stressor-specific effects, the strong compositional overlap between the gut and sediment microbiota highlights the role of sediment ingestion in shaping the intestinal microbiome of *A. japonicus*. This observation echoes parallel findings in *Holothuria leucospilota*, reinforcing the broader ecological pattern that deposit-feeding holothurians acquire a large fraction of their gut microbiota from ingested sediments—a dynamic that may both constrain and enable microbiome responses to environmental stress [58].

### Metabolic dynamics in stress adaptation

The analysis of gut metabolite profiles revealed distinct stress-adaptive strategies through dominant metabolic shifts. The high concentrations of ergothioneine, L-homocystine, and L-pipecolic acid under Con conditions suggest that under normal physiological conditions, gut microbiota synthesize these compounds to maintain intracellular redox homeostasis, regulate microbial osmotic pressure, or participate in host–microbe signaling to stabilize the intestinal microenvironment [59].

The changes in metabolites in the OW group reflect remarkable metabolic reallocation. The accumulation of L-saccharopine indicating enhanced lysine degradation to fuel the tricarboxylic acid (TCA) cycle, and the concurrent reduction in L-pipecolic acid production imply a preferential redirection of lysine derivatives toward energy metabolism rather than osmotic stabilization [60]. This shift aligns with well-documented physiological indicators of thermal stress: elevated temperatures typically increase the host’s ATP demand for stress resistance, and redirecting metabolic flux to the TCA cycle would help compensate for this increased energy requirement, even if it comes at the cost of some homeostatic functions [61, 62]. For example, studies revealed that Pacific oyster (*C. gigas*) larvae under elevated temperatures allocate more ATP toward protein synthesis, potentially compromising energy supply for other physiological processes [62]. Therefore, under warming conditions, microbes may reallocate resources from osmoregulation and antioxidant metabolite synthesis to energy metabolism and heat stress responses to adapt to elevated temperatures rapidly [63, 64].

OA treatment induced restructuring of nitrogen metabolic, wherein elevated L-aspartic acid production coupled with diminished L-homocystine levels reflects a dual mechanism, namely, the promotion of ammonia excretion and minimization of intracellular ammonia accumulation [65]. Such coordinated adjustments would critically mitigate pH-induced toxicity. Among microbial communities, those in the OAW group elicited the most complex response, with the coexisting accumulation of taurine, hydroxyectoine, L-glutamic acid, and γ-glutamyl peptides. This multipronged adaptation demonstrates microbial capacity to activate antioxidant systems, maintain energy flux, and modify metabolic networks simultaneously under compounded stressors [66, 67]. The differential metabolite changes between the Con and treatment groups collectively and consistently point to stress-specific resource allocation: thermal stress favors catabolic energy production and acidification necessitates nitrogen homeostasis, whereas combined stressors demand integrated metabolic flexibility. While shifts in gut microbiome composition likely contribute to these metabolite changes, we acknowledge that physiological adjustments in the host itself may also play a role in shaping the changes in metabolite profiles.

### Microbiota–metabolite interplay in stress resilience

Critically, the observed metabolic and microbial community changes correlate with the interplay between microbial community structure and metabolic outputs. Our metabolomics data not only revealed stress-specific shifts in amino acid derivatives but are further corroborated by parallel 16S rRNA sequencing, which identified the taxonomic rearrangements potentially linked to these metabolic trends. The dominance of Firmicutes in the OAW group—a phylum renowned for its metabolic versatility and stress tolerance—aligns with the upregulation of methylated aspartate derivatives (*N*-methyl-aspartic acid) and γ-glutamyl peptides [68]. Jaiswal et al. (2022) investigated the metabolic pathways of γ-glutamyl peptides in Cyanobacteria through metabolomic analysis and ^13^C-labeling, and revealed significant accumulation of these peptides, suggesting their potential role as amino acid reservoirs for oxidative stress response [66]. Therefore, these taxa may employ posttranslational modifications to stabilize enzymes under combined acidification and warming, thereby sustaining critical metabolic pathways. Conversely, the depletion of Bacteroidetes in OA, which typically degrades complex polysaccharides, correlates with the suppression of L-homocystine, a sulfur-rich metabolite that is dependent on cross-phylum syntropy [55, 69]. This phenomenon suggests that disruptions to microbial interactions driven by pH (e.g. reduced cross-feeding) may constrain sulfur cycling [70]. These findings collectively position microbial community dynamics not merely as bystanders but as factors associated with metabolic reprogramming, wherein taxonomic resilience (e.g. Firmicutes dominance) enables functional redundancy under multifactorial stresses [71].

In conclusion, this study demonstrates correlated changes in the gut microbiota and metabolites of temperate sea cucumbers in response to OA and OW. The gut microbial community structure shifts toward configurations favoring growth under single stressors—a pattern accompanied by metabolic compound shifts that reflect trade-offs between energy allocation and stress protection. Under combined OAW stress, the microbial community shifts toward stress-tolerant taxa, such as Firmicutes, while metabolic profiles show increased synthesis of protective metabolites, such as *N*-methyl-aspartic acid and γ-glutamyl peptides. These metabolic changes stabilize cellular functions, enhance redox homeostasis, and may help the host withstand extreme physicochemical conditions. By linking microbial community restructuring to metabolite changes, this study provides insights into the mechanisms underlying the ability of temperate sea cucumbers to respond to climate change stressors.

## Supplementary Material

3_supporting_information_ycaf188

## Data Availability

The 16S rRNA gene sequencing data generated in this study have been deposited in the NCBI BioProject database under the accession number PRJNA1270743. All other relevant data supporting the findings of this study are included in the main manuscript and its supplementary materials.
