# Supplementary material for: Gut microbial community plasticity as a climate shield mediating sea cucumber resilience to ocean acidification and warming
Source: ISME Commun. 2025 Oct 23;5(1):ycaf188. doi: 10.1093/ismeco/ycaf188 (PMC12596727; doi:10.1093/ismeco/ycaf188)
Supplement: 3_supporting_information_ycaf188 [file 3_supporting_information_ycaf188.pdf]

Supporting Information for

Gut microbial community plasticity as a climate shield mediating sea cucumber resilience to ocean acidification and warming

Encui Shan [a, c, d], Zhenglin Yu [a, b], Xiao Cong [a, b], Chaowei Hou [a, b, c, d], Xueying Guo [a, c, d], Lei Pang [a, c, d], Jianmin Zhao [a, b, d], Qing Wang [a, b, d], Xiutang Yuan [a, b*]

[a] Research and Development Center for Efficient Utilization of Coastal Bioresources, Yantai Institute of Coastal Zone Research, Chinese Academy of Sciences, Yantai 264003, P. R. China

[b] Laboratory for Marine Biology and Biotechnology, Qingdao Marine Science and Technology Center, Qingdao 266000, P. R. China

[c] University of Chinese Academy of Sciences, Beijing 100049, P.R. China

[d] Muping Coastal Environment Research Station, Yantai Institute of Coastal Zone Research, Chinese Academy of Sciences, Yantai 264003, P. R. China

*Corresponding author:

Dr. Xiutang Yuan

Yantai Institute of Coastal Zone Research, Chinese Academy of Sciences

Chunhui Road No. 17, Laishan District, Yantai City, Shandong Province, 264003, P. R. China

Catalog:

Figure S1. Field sampling site (a) and the cultivation conditions of mesocosm (b).

Figure S2. Rarefaction Curve of samples.

Table S1. Relative abundance (%) of the top 10 taxonomic of microbial communities at the phylum levels of different groups.

Table S2. Relative abundance (%) of the top 10 taxonomic of microbial communities at the family levels of different groups.

Table S3. Statistical analysis of similarity (ANOSIM) of bacterial communities among different treatments. Significant differences ($p < 0.05$) are shown in bold.

Table S4. Variation in *A. japonicus* gut microbiome and sediment alpha diversity under different treatments.

Table S5. Qualitative and quantitative of amino acid metabolites in the gut microbiota of each treatment group.

Table S6. Screening of differential metabolites between control group (Con) and environmental stress group (OA/OW/OAW).

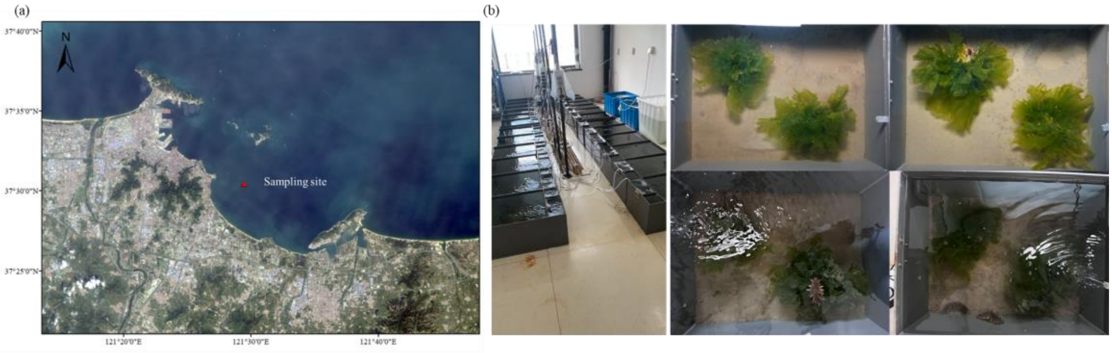

Figure S1. Field sampling site (a) and the cultivation conditions of mesocosm (b).

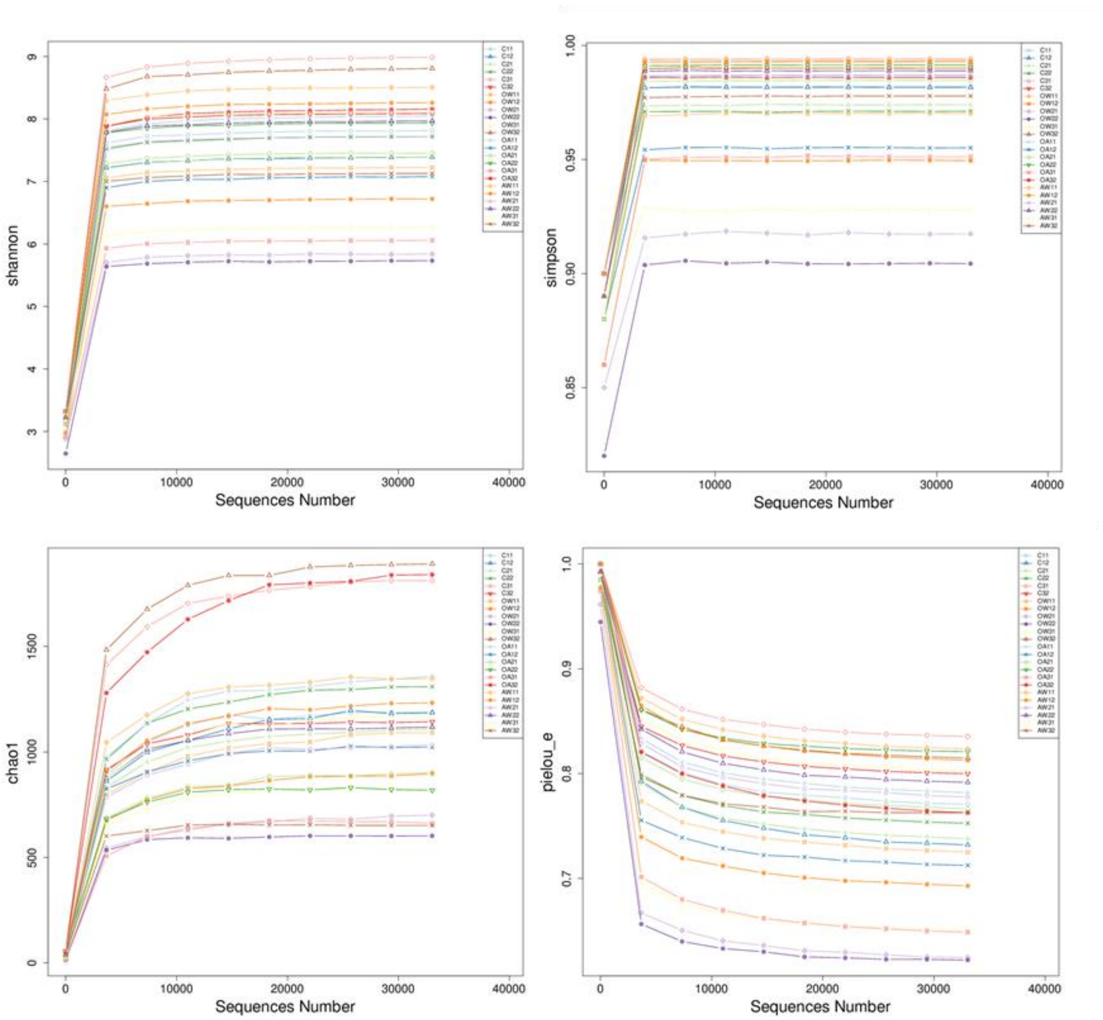

Figure S2. Rarefaction Curve of samples.

Note: The horizontal axis represents the sequencing number, and the vertical axis represents the corresponding alpha diversity index. When the curve tends to flatten, it

indicates that the sequencing data volume is gradually reasonable, and more data volume will not have a significant impact on the alpha diversity index.

Table S1. Relative abundance (%) of the top 10 taxonomic of microbial communities at the phylum levels of different groups.

Phylum	CON	OW	OA	OAW
Proteobacteria	0.504233	0.7883	0.631604	0.636064
Bacteroidota	0.163099	0.071009	0.156484	0.121901
Desulfobacterota	0.0662	0.019628	0.033398	0.041703
Verrucomicrobiota	0.067714	0.014673	0.066811	0.067543
Actinobacteriota	0.058677	0.046719	0.042642	0.066639
Cyanobacteria	0.022534	0.004415	0.005752	0.004269
Acidobacteriota	0.031844	0.010122	0.006741	0.012438
Firmicutes	0.021011	0.004223	0.004435	0.002528
Dependentiae	0.002861	0.005626	0.011701	0.002654
Chloroflexi	0.012221	0.002911	0.003209	0.004057
Others	0.049606	0.032374	0.037223	0.040204

Table S2. Relative abundance (%) of the top 10 taxonomic of microbial communities at the family levels of different groups.

Family	CON	OW	OA	OAW
Rhodobacteraceae	0.218204	0.17637	0.327586	0.209394
Vibrionaceae	0.045417	0.341729	0.089174	0.084996
Flavobacteriaceae	0.157418	0.064419	0.144127	0.111476
Halieaceae	0.076211	0.109513	0.076166	0.144354
Rubritaleaceae	0.05515	0.010682	0.051512	0.054842
unidentified_Gammaproteobacteria	0.037435	0.033781	0.036572	0.058637
unidentified_Chloroplast	0.021041	0.004153	0.00441	0.003789
Woeseiaceae	0.008401	0.023902	0.011943	0.023483
Desulfocapsaceae	0.026546	0.006938	0.017761	0.016585
Lachnospiraceae	0.014486	0.000434	0.002447	0.000308
Others	0.339691	0.228079	0.238302	0.292136

Table S3. Statistical analysis of similarity (ANOSIM) of bacterial communities among different treatments. Significant differences ($p < 0.05$) are shown in bold.

Group	Sums of Sqs	F.Model	R^2	Pr (>F)
Con.-OW	0.71144(1.26839)	5.60903	0.35935(0.64065)	**0.003**
Con.-OA	0.27283(1.70793)	1.59746	0.13774(0.86226)	0.057
Con.-OAW	0.30841(1.23009)	2.50723	0.20046(0.79954)	**0.01**
OW-OA	0.57992(1.39641)	4.15294	0.29343(0.70657)	**0.009**
OW-OAW	0.41795(0.91857)	4.54998	0.31271(0.68729)	**0.002**
OA-OAW	0.28554(1.35811)	2.10246	0.17372(0.82628)	**0.019**

Table S4. Variation in *A. japonicus* gut microbiome and sediment alpha diversity under different treatments.

	chao1	pielou_e	shannon	simpson
Gut.Con	4459.621	0.721	8.665	0.99
Gut.OW	3466.402	0.624	7.258	0.951
Gut.OA	3845.46	0.704	8.282	0.989
Gut.OAW	3284.984	0.726	8.393	0.992
Sediment.Con	7212.052	0.762	9.599	0.995
Sediment.OW	7515.638	0.76	9.625	0.994
Sediment.OA	6886.686	0.745	9.343	0.994
Sediment.OAW	8771.692	0.775	9.958	0.997

Table S5. Qualitative and quantitative of amino acid metabolites in the gut microbiota of each treatment group.

Metabolite name	CON	OW	OA	OAW
Glycine	3489.119	3107.373	3128.878	5607.858
L-Alanine	31665.06	34018.47	29639.03	51951.43
Beta-Alanine	2443.258	2488.939	1753.687	2438.697
N-Methylalanine	37.87701	18.31846	18.31435	20.91885
Dimethylglycine	62.74717	10.39072	4.254217	11.80253
Creatinine	100.9843	45.35795	124.5011	147.7272
L-Proline	12870.64	11033.91	12910.66	18734.42
L-Valine	5021.713	4346.638	4822.159	8299.37
Norvaline	1419.122	1273.074	1149.147	1639.391
Betaine	527.8358	374.7709	677.7276	252.5753
N-Methyl-4-aminobutyric acid	256.342	110.8416	193.9129	214.4516
L-Threonine	15431.18	14797.27	11276.46	31186.37
Pyroglutamic acid	30.84267	40.62047	33.44154	49.06518
L-Pipecolic acid	6291.363	1717.259	4101.199	940.7902
4-Hydroxyproline	139.2788	218.2071	167.2617	208.7979
5-Aminolevulinic acid	285.9831	104.1382	111.7543	148.9196
L-Isoleucine	9162.277	8635.317	8600.18	13387.66
L-Leucine	21519.85	18739.62	15457.99	29660.37
L-Asparagine	11070.57	15902.18	13508.69	20276.35
Glycylglycine	80.58998	142.4328	109.8232	210.3923
L-Ornithine	266.2946	372.8323	191.9666	409.915
Ectoine	92.70049	116.0131	70.57548	107.7861
4-acetamidobutanoate	25.48948	61.78076	27.84391	29.95631
4-guanidinobutanoate	1056.87	987.6475	1977.99	1247.542
L-Glutamine	59268.69	39516.29	78653.22	86587.35
L-Lysine	39966.89	25562.75	16100.81	41324.24
L-Glutamic acid	1051327	896604.4	1860584	2157256
N-Methyl-aspartic acid	18636.92	18899.41	78111.85	57400.45
L-Methionine	6130.584	3583.832	1975.605	3735.899
L-Histidine	1268.14	1326.064	970.727	2663.918
N-Acetylproline	7.821766	7.701273	14.40783	10.93484

Hydroxyectoine	39165.07	16330.95	23656.22	45808.04
2-Aminoadipic acid	425.1239	376.5463	295.4246	423.2471
L-Phenylalanine	6610.573	4972.224	3896.671	7162.84
methionine sulfoxide	95304.74	58395.61	47927.5	164835.3
3-Methylhistidine	147.636	132.6177	194.2627	298.0307
1-Methylhistidine	4.975204	2.403744	7.769407	1.627763
Glycylproline	1548.11	646.3713	3724.118	4183.648
L-Theanine	465.5826	212.0769	454.4208	396.2119
L-Arginine	3593.199	3735.098	1911.081	4028.07
Citrulline	13082.65	7278.455	5525.722	15661.39
Nicotinuric acid	379716.2	180046.3	50049.19	643636.4
L-Tyrosine	87289.11	34947.04	18147.41	138485.2
N6-Acetyllysine	1786.62	939.0267	1675.781	2937.488
N-Alpha-acetyllysine	228.7255	46.65951	467.9265	220.5199
homoarginine	6.252731	11.61086	5.0759	3.801467
Methyl hippurate	0.132496	0.149166	0.328	0.108314
L-Histidine trimethylbetaine	465.3192	739.1919	822.2163	696.3719
Asymmetric dimethylarginine	3818.884	2401.104	4482.107	4625.808
L-Tryptophan	1299.475	285.1716	1769.198	514.8773
N-acetylphenylalanine	4.340194	3.793714	2.010071	4.980415
N-acetylarginine	16.76394	10.72212	15.74561	5.912935
O-Succinyhomoserine	32985.45	15700.48	88128.47	50233.89
Ergothioneine	679480.6	154532	561630.7	390886
L-Leucyl-L-Valine	25085.17	42911.36	102385.8	36811.19
N-Acetyl-L-tryptophan	4639.071	7176.447	5052.62	10037.62
gamma-Glutamyl-valine	10457.47	3862.237	7681.523	13650.19
Phenylalanylvaline	8.375549	44.1439	20.1678	26.34138
L-Homocystine	14790.89	4425.984	3327.837	3160.445
L-Saccharopine	12086.06	13988.75	9465.243	13608.71
gamma-Glutamyl-methionine	1949.844	1180.814	907.1175	2881.337
Ophthalmic acid	212.4033	231.8838	1339.476	946.1216
gamma-Glutamylphenylalanine	387.5157	281.7602	172.6998	1119.855
Phenylalanyltryptophan	389.6712	762.2856	997.1703	586.457
3,5-Diiodo-tyrosine	11.22145	11.12552	15.32838	11.96059
Liothyronine	16.36594	28.01061	25.16252	26.96797

N-Formylglycine	30.33039	26.10082	56.07372	43.78265
L-Serine	22130.33	27845.78	29238.63	34590.31
Acetylglycine	28.92541	14.00294	23.0903	22.17765
Taurine	25750.02	16272.17	11447.69	28284.02
N-Acetyl-L-alanine	1339.337	431.6115	462.7779	609.2671
Propionylglycine	27.30837	17.72369	15.21875	16.91361
N-Carbamoylsarcosine	11.71064	33.20104	17.89926	12.01174
L-Aspartic acid	265828.2	337386.6	553390.3	788300.2
N-Acetylserine	710.5554	617.1823	616.6816	613.22
L-Cysteinesulfinic acid	181.5027	234.1245	159.8794	311.3581
L-Dihydroorotic acid	3.741524	3.706646	3.670014	5.157287
Isovalerylglycine	5.003852	3.745959	3.478195	7.154482
N-acetylvaline	34.56854	45.05601	58.30277	53.23362
N-Formyl-L-aspartate	841.6514	913.2218	1684.317	1835.232
N-acetylleucine	9.61734	9.320022	10.73952	9.689572
N-Acetylasparagine	2.174648	3.930615	7.17984	3.69439
N-Acetyl-L-aspartic acid	5.967726	4.590033	6.196776	7.730387
N-Carbamoyl-DL-aspartic acid	2.415992	8.497009	16.47294	7.54135
N-formylmethionine	25.15926	3.923931	6.963545	5.918183
N-Acetylglutamine	8.81766	8.976038	61.6263	18.98853
N-Acetyl-L-glutamic acid	185.423	228.507	210.7013	196.8339
N-Carbamylglutamate	11.31634	21.13598	20.41874	19.24182
N-acetylcitrulline	6.189141	7.514744	7.859278	6.75889
Gama-glutamylalanine	114.6308	259.0116	213.5285	346.7594
gamma-glutamylglutamate	5212.389	8602.194	8940.001	13496.26

Table S6. Screening of differential metabolites between control group (Con) and environmental stress group (OA/OW/OAW).

	Metabolite name	Con	OAW	FC value	*P* value	log_2FC	up vs. down
Con vs. OAW	L-Pipecolic acid	6291.36	940.79	6.687317	0.01	2.74142	up
	N-Methyl-aspartic acid	18636.92	57400.45	0.324682	0.02	-1.62289	down
	L-Homocystine	14790.89	3160.45	4.680002	0.03	2.22650	up
	L-Aspartic acid	265828.20	788300.20	0.337217	0.01	-1.56825	down
	N-Carbamoyl-DL-aspartic acid	2.42	7.54	0.320365	0.02	-1.64220	down
Con vs. OA	Metabolite name	Con	OA	FC value	*P* value	log_2FC	up vs. down
	L-Homocystine	14790.89	3327.84	4.444595	0.03	2.15205	up
Con vs. OW	Metabolite name	Con	OW	FC value	*P* value	log_2FC	up vs. down
	L-Pipecolic acid	6291.36	1717.26	3.663608	0.02	1.87326	up
	Ergothioneine	679480.60	154532.00	4.397021	0.04	2.13652	up
